# GRB7 is an oncogenic driver and potential therapeutic target in oesophageal adenocarcinoma

**DOI:** 10.1002/path.5528

**Published:** 2020-09-15

**Authors:** Jovana R Gotovac, David SH Liu, Michael J Yates, Julia V Milne, Arthi A Macpherson, Kaylene J Simpson, Guy D Eslick, Catherine Mitchell, Cuong P Duong, Wayne A Phillips, Nicholas J Clemons

**Affiliations:** ^1^ Division of Cancer Research Peter MacCallum Cancer Centre Melbourne Victoria Australia; ^2^ Sir Peter MacCallum Department of Oncology The University of Melbourne Parkville Victoria Australia; ^3^ Nepean Clinical School The University of Sydney Kingswood New South Wales Australia; ^4^ Department of Pathology Peter MacCallum Cancer Centre Melbourne Victoria Australia; ^5^ Department of Surgery (St Vincent's Hospital) The University of Melbourne Parkville Victoria Australia

**Keywords:** oesophageal adenocarcinoma (OAC), GRB7, oncogene, HER2/ERBB2, trastuzumab, targeted therapy, receptor tyrosine kinase, reverse phase protein array (RPPA)

## Abstract

Efficacious therapeutic approaches are urgently needed to improve outcomes in patients with oesophageal adenocarcinoma (OAC). However, oncogenic drivers amenable to targeted therapy are limited and their functional characterisation is essential. Among few targeted therapies available, anti‐human epidermal growth factor receptor 2 (HER2) therapy showed only modest benefit for patients with OAC. Herein, we investigated the potential oncogenic role of growth factor receptor bound protein 7 (GRB7), which is reported to be co‐amplified with HER2 (ERBB2) in OAC. GRB7 was highly expressed in 15% of OAC tumours, not all of which could be explained by co‐amplification with HER2, and was associated with a trend for poorer overall survival. Knockdown of GRB7 decreased proliferation and clonogenic survival, and induced apoptosis. Reverse phase protein array (RPPA) analyses revealed a role for PI3K, mammalian target of rapamycin (mTOR), MAPK, and receptor tyrosine kinase signalling in the oncogenic action of GRB7. Furthermore, the GRB7 and HER2 high‐expressing OAC cell line Eso26 showed reduced cell proliferation upon GRB7 knockdown but was insensitive to HER2 inhibition by trastuzumab. Consistent with this, GRB7 knockdown *in vivo* with an inducible shRNA significantly inhibited tumour growth in cell line xenografts. HER2 expression did not predict sensitivity to trastuzumab, with Eso26 xenografts remaining refractory to trastuzumab treatment. Taken together, our study provides strong evidence for an oncogenic role for GRB7 in OAC and suggests that targeting GRB7 may be a potential therapeutic strategy for this cancer. © 2020 The Authors. *The Journal of Pathology* published by John Wiley & Sons, Ltd. on behalf of The Pathological Society of Great Britain and Ireland.

## Introduction

Cancer of the oesophagus remains the sixth major cause of cancer‐related deaths worldwide [1]. There are two major histological subtypes of oesophageal cancer: oesophageal squamous cell carcinoma (OSCC) and OAC. Although the most common subtype worldwide is OSCC, the incidence of OAC has risen rapidly over the last four decades in North America, Europe, and Australia, whereas the incidence of OSCC has decreased [2, 3]. The prognosis for OAC is poor, with 5‐year survival ranging from 14% to 22% [4, 5]. Despite the application of multimodality therapy, which consists of chemotherapy, radiotherapy, and surgery, the majority of patients are either unresectable, experience early relapse, or develop distant metastatic disease with limited treatment options. Hence, there is an urgent need for new therapeutic strategies.

In an era of effective molecular‐targeted therapy in treating solid cancers such as breast [6, 7] and lung cancers [8], OAC is still lacking defined oncogenic drivers amenable to therapeutic targeting. The only two FDA‐approved targeted therapies for the treatment of OAC – trastuzumab (Herceptin), targeting human epidermal growth factor receptor 2 (HER2; ERBB2), and ramucirumab, targeting vascular endothelial growth factor receptor 2 (VEGFR2) – have shown only modest survival benefit [9, 10]. This may be explained partly by the activation of multiple oncogenic pathways that maintain tumour survival and mediate therapeutic resistance in this disease [11]. Therefore, inhibiting intersecting signalling pathways may have greater therapeutic efficacy.

The *HER2* proto‐oncogene is positioned within the 17q12 amplicon and its amplification and overexpression have been frequently associated with gastrointestinal carcinogenesis [12, 13, 14]. However, further molecular characterisation of the 17q12 amplicon has shown that this region also contains other genes frequently amplified with *HER2*, including *GRB7* [15, 16].

GRB7 is an SH2‐domain‐containing adaptor molecule that mediates cellular signalling through interaction with multiple receptor tyrosine kinases and their downstream partners [17]. In this way, GRB7 is a central node that connects multiple potential oncogenic drivers to downstream signalling pathways and thus, represents an attractive therapeutic target. High GRB7 expression is associated with decreased survival in patients with breast cancer [18], whereas overexpression of GRB7 and its variant (GRB7v) is correlated with high‐grade ovarian cancers [19]. Overall, the lack of preclinical evidence with regard to the functional role of GRB7 amplification and/or overexpression in oesophageal cancer is preventing the identification of any potential therapeutic benefits of targeting GRB7 in this disease.

Herein, for the first time, we report the frequency of GRB7 protein expression levels within an OAC patient cohort and correlation with survival outcome. Furthermore, we assayed the functional effects of manipulating GRB7 expression levels in *in vitro* cell line models of OAC. Importantly, we demonstrated the therapeutic value of inhibiting GRB7 in OAC xenografts. In summary, our body of work highlights the potential oncogenic role and therapeutic significance of GRB7 in OAC.

## Materials and methods

### Oesophageal cancer research cohort

Use of patient samples in this study was approved by the Human Research Ethics Committee of the Nepean Blue Mountains Local Health District. All patients provided written informed consent prior to recruitment. Tumour microarrays (TMAs) of tumours from an OAC patient cohort were assessed for GRB7 positivity. Information about this patient cohort, including detailed clinico‐pathological data and HER2 status, has been published previously [20]. Sufficient tissue and clinico‐pathological data were available for 88 patients.

### Histology and immunohistochemistry (IHC)

**Figure 1:**
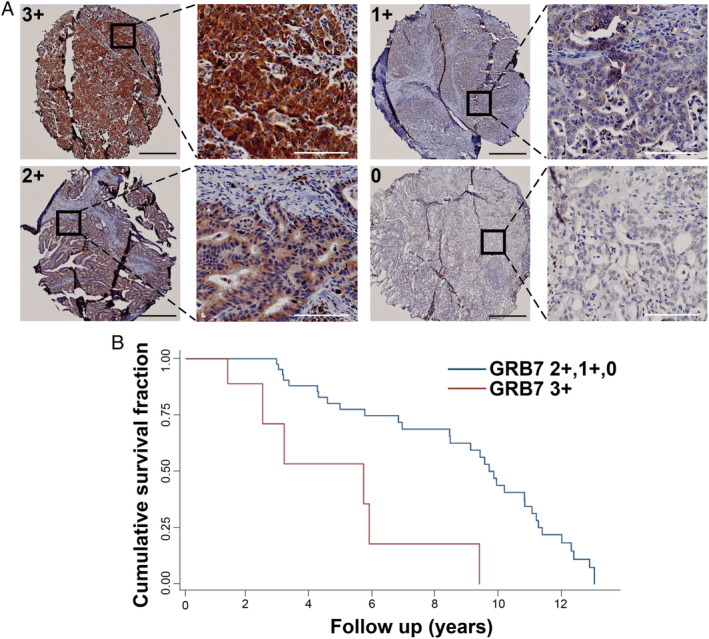
Overexpression of GRB7 correlates with poor OAC patient outcome. (A) Representative images of OAC biopsy cores used as standard for GRB7 IHC scoring system (3+, 2+, 1+ and 0), respectively. Black scale bars = 500 μm; white scale bars = 100 μm. (B) Kaplan–Meier curves demonstrating the effect of GRB7 high expression (IHC 3+, red curve) versus GRB7 equivocal/low/negative expression (2+, 1+, 0, blue curve) on overall survival. Logistic regression analysis was used to compare survival between 13 OAC patients with GRB7‐high tumours and 75 patients with GRB7‐equivocal/low/negative tumours.

TMA sections were obtained from formalin‐fixed, paraffin embedded (FFPE) tissue blocks and stained with H&E for histological features or with anti‐GRB7 antibody (supplementary material, Table S1). A detailed IHC protocol is presented in supplementary material, Supplementary materials and methods. Images were captured using a VS‐120 microscope (Olympus, Tokyo, Japan), and two independent researchers (JRG and NJC) performed scoring. In co‐operation with an expert pathologist (CM), the final consensus score was obtained. GRB7 IHC was assessed as 3+/positive (strong complete cytoplasmic and basolateral reactivity), 2+/equivocal (weak‐to‐moderate complete cytoplasmic and basolateral reactivity), 1+/low (faint incomplete cytoplasmic reactivity, or 0/negative (no cytoplasmic reactivity) (Figure 1A). Only staining in tumour cells was scored and homogeneous staining was observed in each core across the tumour cell portion.

### Cell lines and culture

Detailed information about the cell lines and cell culture conditions is provided in supplementary material, Supplementary materials and methods. All cell lines were authenticated by short tandem repeats (STR) analyses using the PowerPlex® 16 System (Promega, Madison, WI, USA) and confirmed mycoplasma‐free by PCR analyses (Cerberus Sciences, Scoresby, Australia).

### Transient GRB7 knockdown

Cells were transfected with 40 nm ON‐TARGETplus non‐targeting pool or siNTC (GE Dharmacon, Lafayette, CO, USA; D‐001810‐10) or *GRB7* siRNA (siGENOME SMARTpool, GE Dharmacon; M‐012701‐01) using Lipofectamine RNAiMax solution (Thermo Fisher Scientific, Carlsbad, CA, USA) according to the manufacturer's instructions. The GRB7 siRNA SMART pool targets exons 4, 5, 11, and 14 of the *GRB7* transcript. Target sequences are provided in supplementary material, Table S2.

### GRB7 knockdown using shRNA

GRB7‐specific shRNAs (supplementary material, Table S3) and sh*Renilla* control (sh Control) were cloned into the doxycycline‐inducible LT3GECIR lentiviral expression vector containing the optimised miR‐E backbone [21]. The mCherry and turbo‐GFP reporters were used to monitor transduction efficiency and induction of the shRNA, respectively. OE19 and Eso26 cells were induced with doxycycline (Merck, Kenilworth, NJ, USA; 2 μg/ml) for 48 h before cytometric sorting (BD Fusion5™; BD Biosciences, Franklin Lakes, NJ, USA) to isolate mCherry‐ and GFP‐positive cells.

### Reverse transcription‐quantitative PCR (RT‐qPCR)

RNA extraction and RT‐qPCR were as described previously [22]. Primers used for RT‐qPCR are summarised in supplementary material, Table S4.

### Assays for proliferation, cell viability, clonogenicity, migration, apoptosis, and cell cycle

The assays used to test the effect of different treatments on cellular proliferation (Incucyte FLR, Essen BioScience, Michigan, USA) [23], and viability [AlamarBlue®, (Thermo Fisher Scientific) [24] or CellTiter‐Glo® (Promega)] [25], clonogenic survival [26], migratory capacity [27], apoptosis [28], and cell cycle distribution [29] have been described previously. Specific assay conditions used in this study are described in supplementary material, Supplementary materials and methods.

### Western blotting analysis

Cells and tissues were lysed and prepared as specified in supplementary material, Supplementary materials and methods. The antibodies used are listed in supplementary material, Table S1.

### GRB7 overexpression

*GRB7* was expressed ectopically in FLO‐1, OACP4C, and OE33 cells using the Precision LentiORF pLOC lentiviral vector containing turbo‐GFP as a reporter gene (OHS5898‐202620211, GE Dharmacon). The ectopic expression of turbo‐RFP in the same vector was used as a control. GFP‐positive FLO‐1, OACP4C, and OE33 cells were sorted by FACS.

### Tumour xenografts

All animal experiments were conducted in accordance with the *National Health and Medical Research Council Australian Code of Practice for the Care and Use of Animals for Scientific Purposes* and approved by the Peter MacCallum Cancer Centre (PMCC) Animal Experimentation Ethics Committee.

NOD‐SCID IL‐2RγKO (NSG) mice were obtained from the Garvan Institute of Medical Research (Sydney, Australia) or bred in‐house. To generate OE19 and Eso26 cell line xenografts (CLXs), 5 × 10^6^ cells were resuspended in 100 μl of 1:1 ice‐cold phosphate‐buffered saline (PBS) and growth factor reduced Matrigel® Matrix (Corning, Corning NY, USA) and kept on ice. Cells were injected subcutaneously into the right flanks of 6‐ to 8‐week‐old female NSG mice. When tumours reached 150 mm^3^ volume (calculated using the formula length × width^2^/2), mice were randomised into groups of 5–8 for treatments. Concomitantly with vehicle, doxycycline, and/or trastuzumab treatments, subcutaneous tumour volume was measured with callipers twice per week up to the end of the experiment. All mice were euthanised when subcutaneous tumours reached ≥ 1500 mm^3^ or at the first signs of ill health or discomfort, apart from six mice that were euthanised earlier at 15, 25 or 30 days to determine GRB7 knockdown efficiency *in vivo*.

### Reverse phase protein array (RPPA)

Protein lysates were prepared from treated tissue culture cells in CLB1 buffer (Zeptosens/Bayer, Leverkusen, Germany), and protein was quantified using a Pierce™ Coomassie Plus Protein Assay Kit (Thermo Fisher Scientific) (*n* = 3 per treatment). Details of the RPPA protocol are given in supplementary material, Supplementary materials and methods.

### Statistics

Data were analysed using unpaired *t*‐tests to compare two groups of interest. For analyses of three or more groups, parametric one‐way ANOVA with Dunnett's multiple comparisons *post hoc* tests was performed. Statistical analyses were performed using Prism 7 (GraphPad, San Diego, CA, USA). Contingency table analysis with a chi‐squared test was used to compare GRB7 3+, 2+, 1+, and 0 IHC scoring with HER2 positivity (HER2^+^ tumours). Kaplan–Meier curves were generated for overall survival, defined as the time from diagnosis to death from any cause. Statistics for RPPA protein RFI values between treatment and control samples was obtained with Welch's *t*‐test. For analyses of the CLX tumour volume over the time course of the treatment, we compared groups of growth curves between different treatments in OE19 and Eso26 xenografts by using a CGGC permutation test. This test performs permutation tests of the differences between groups of the growth curves for the full‐time course of the treatment [30, 31]. For all statistical analyses, *p* < 0.05 was considered statistically significant.

## Results

### GRB7 expression status in OAC patient samples and correlation with survival outcome

We evaluated the expression of GRB7 in TMAs from 88 patient samples [20]. Representative images of OAC biopsy cores for different scoring intensities including 3+, 2+, 1+, and 0 are shown in Figure 1A and the proportion of samples with each intensity score is summarised in Table 1. We also examined the correlation between GRB7 expression and HER2 status (previously reported for this cohort) [20] as the *GRB7* gene is located within the 17q12 amplicon and is reported to be co‐amplified and overexpressed with *HER2* in OAC [32]. While high GRB7‐expressing tumours (IHC 3+) were more likely to be HER2‐positive (*p* = 0.03) compared with equivocal/low/negative GRB7‐expressing tumours (IHC 2+, 1+, 0), nearly 70% of high GRB7‐expressing tumours were HER2‐negative. This would suggest that high GRB7 expression was not just the result of co‐amplification with HER2 and that other mechanisms for increasing GRB7 expression exist. Importantly, there was a trend for worse outcomes in patients with high tumour GRB7 expression (Figure 1B). In continuation, we next functionally characterised the effect of genetic GRB7 knockdown alone or in combination with HER2 inhibition in OAC cells.

**Table 1:** IHC scoring for GBR7.

GRB7 intensity score	OAC tumours	HER2^+^ *	HER2^–^
	*n* (%)	*n* (%)	*n* (%)
0	28 (31.8)	2 (7.1)	26 (92.9)
1+	28 (31.8)	1 (3.6)	27 (96.4)
2+	19 (21.6)	1 (5.3)	18 (94.7)
3+	13 (14.8)	4 (30.8)^†^	9 (69.2)
Total	88 (100)	8 (9.1)	80 (90.9)

* HER2^+^: either 3+ IHC staining or 2+ IHC staining and SISH positivity (six or more *HER2* gene copies and/or a HER2/chr17 ratio greater than 2).

^†^ *p* = 0.03, chi‐squared test.

### GRB7 knockdown has an advantage over anti‐HER2 therapy in decreasing cellular proliferation *in vitro*

**Figure 2:**
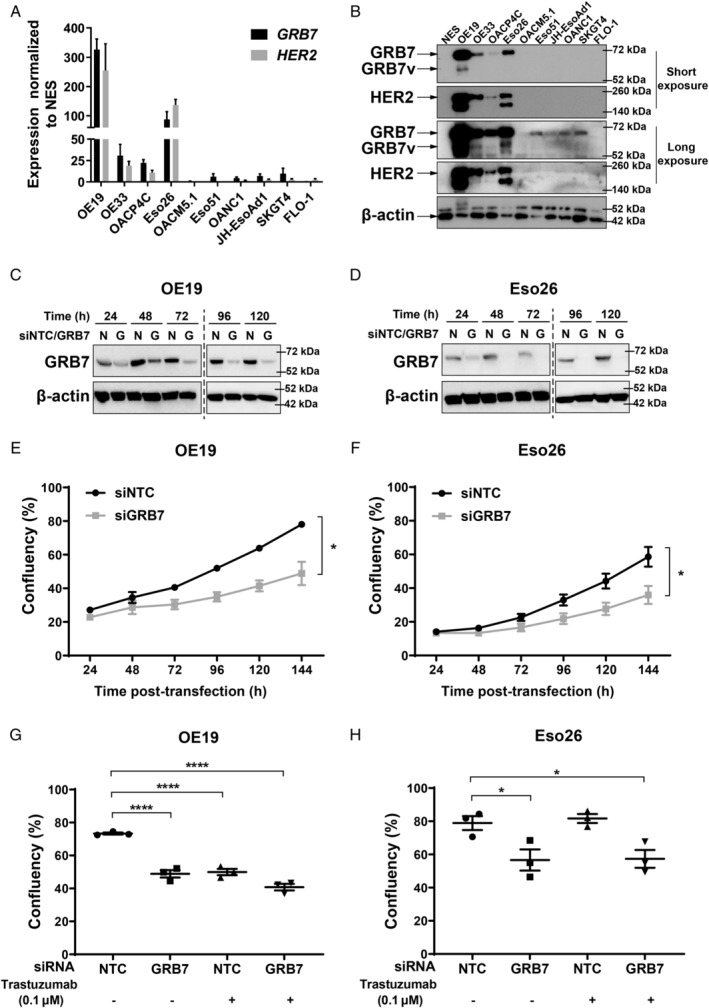
Trastuzumab‐resistant Eso26 cells respond to the depletion of GRB7 by suppression of cell proliferation. Basal expression of GRB7 and HER2 in a panel of ten human OAC cell lines and a normal oesophageal squamous cell line (NES) determined by RT‐qPCR (A) and western blotting (B). Representative western blots imaged at two different exposure times are shown, with arrows indicating position of the protein size. GRB7 protein levels in OE19 (C) and Eso26 (D) cells following knockdown with GRB7 siRNAs (siGRB7 or G) compared with non‐targeting control siRNA (siNTC or N). Western blots were performed 24–120 h after transfection. The dashed dividing line indicates separate western blots. Cell confluency measured using an Incucyte optical scanner from 24 to 144 h after transfection with GRB7 siRNA or siNTC in OE19 (E) and Eso26 (F) cells. Cell confluency of OE19 (G) and Eso26 (H) cells 144 h after transfection with siNTC or siGRB7, alone or in combination with trastuzumab (0.1 μm). Bars represent mean ± SEM from three independent experiments (no error bars indicate SEM less than size of symbol). Statistics: (E, F) Unpaired *t*‐test, *p < 0.05 for the last time point. (G, H) One‐way ordinary ANOVA with Dunnett's multiple comparisons post‐test, *p < 0.05, ****p < 0.0001.

The mRNA transcript and protein expression levels of GRB7 and HER2 were investigated across a panel of ten OAC cell lines and in a normal oesophageal squamous cell line (NES). *GRB7* and *HER2* mRNA expression was significantly higher in OE19, OE33, OACP4C, and Eso26 tumour cell lines than in non‐cancerous NES cells (Figure 2A), consistent with *GRB7* and *HER2* gene amplification in these cell lines (supplementary material, Table S5). Similarly, high GRB7 and HER2 protein expression levels were detected in OE19 and Eso26 cell lines (Figure 2B), while OE33, OACP4C, and the other cell lines expressed GRB7 and HER2 protein at lower levels (Figure 2B). OE19 and Eso26 cells also show the presence of a smaller molecular weight GRB7 protein, which is likely to be GRB7 variant protein [19, 33]. To evaluate the functional contribution of high GRB7 expression, GRB7 knockdown was performed using siRNA targeting exons common to both full‐length and variant GRB7. Efficient knockdown of *GRB7* mRNA (supplementary material, Figure S1A,B) and protein levels (Figure 2C,D; the dashed line indicates separate blots) significantly decreased cell proliferation in GRB7 high‐expressing OE19 and Eso26 cells (Figure 2E,F). In contrast, GRB7 knockdown in cell lines with low GRB7 protein expression levels (NES, OANC1, OE33, and OACP4C) did not affect cellular proliferation compared with control siRNA (supplementary material, Figure S1C–F).

Given that GRB7 may act as a central node for oncogenic signalling and is commonly overexpressed in OAC (Table 1), including cell line models (Figure 2A,B), GRB7 presents a rational therapeutic target as an alternative, or in addition, to current targeting of HER2. To address this hypothesis, we compared the effect of GRB7 knockdown and HER2 inhibition, alone or in combination, in OE19 and Eso26 cells. Based on dose response curves to trastuzumab (0.0001–10 μm), only OE19 was found to be sensitive to trastuzumab alone, with maximum efficacy reached at the concentration 0.1 μm (results not shown). Importantly, in OE19 cells, GRB7 knockdown or trastuzumab only treatment significantly decreased cellular proliferation (Figure 2G). However, there was no additional growth inhibitory effect when combining GRB7 knockdown with trastuzumab. In contrast, in HER2‐amplified Eso26 cells, trastuzumab alone did not result in any growth inhibitory effect *in vitro*, whilst GRB7 knockdown significantly decreased cellular proliferation (Figure 2H). Similar to OE19 cells, the addition of trastuzumab to GRB7 knockdown did not result in further growth inhibition. These results support our hypothesis that GRB7 functions as a central node for oncogenic signalling and that its inhibition may achieve a wider therapeutic applicability than targeting HER2.

### GRB7 knockdown induces apoptosis in OAC cells

**Figure 3:**
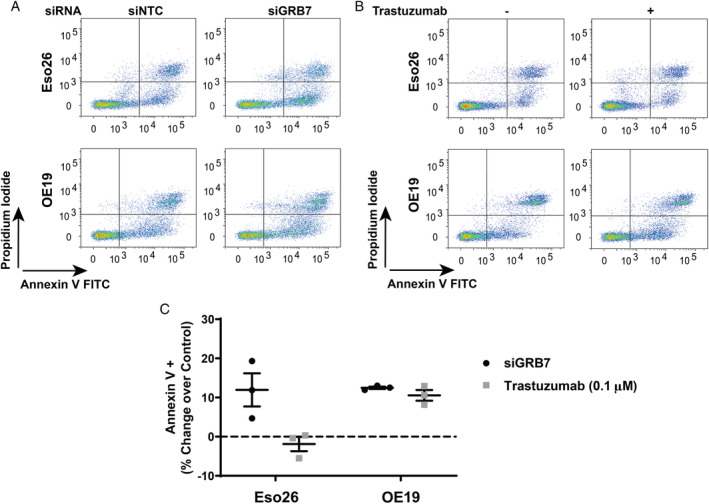
GRB7 knockdown induces apoptosis in high HER2‐expressing but trastuzumab‐resistant Eso26 cells. Representative Annexin V‐FITC/propidium iodide (PI) FACS plots of Eso26 and OE19 cells transfected with siNTC or siGRB7 for 144 h (A) or treated with vehicle or trastuzumab (0.1 μm) for 120 h (B). (C) Quantification of Annexin‐V‐positive cells in Eso26 and OE19 cells treated with siGRB7 and trastuzumab (0.1 μm) compared with siNTC and vehicle, respectively. Bars represent mean ± SEM for three independent experiments.

We next assessed whether reduced cell growth was due to cell cycle arrest and/or apoptosis following GRB7 knockdown or HER2 inhibition. GRB7 knockdown or HER2 inhibition did not lead to G1/S or G2/M arrest in Eso26 or OE19 cells (supplementary material, Figure S2A,B). However, an increase in the subG0 fraction was observed following siGRB7 knockdown in both cell lines or trastuzumab treatment in OE19 cells (supplementary material, Figure S2C), suggesting that these cells are undergoing apoptosis. To clarify whether GRB7 knockdown or HER2 inhibition induces apoptosis, we quantified the percentage of Annexin‐V‐positive cells at 144 h after GRB7 knockdown or 120 h following treatment with trastuzumab. In both cell lines, GRB7 knockdown led to apoptosis (Figure 3A,C). Consistent with other results, trastuzumab treatment induced apoptosis in OE19 but not Eso26 cells (Figure 3B,C). Further, we next aimed to decipher molecular mechanisms of GRB7 oncogenic function.

### Deciphering GRB7 mechanism of oncogenic signalling transduction

**Figure 4:**
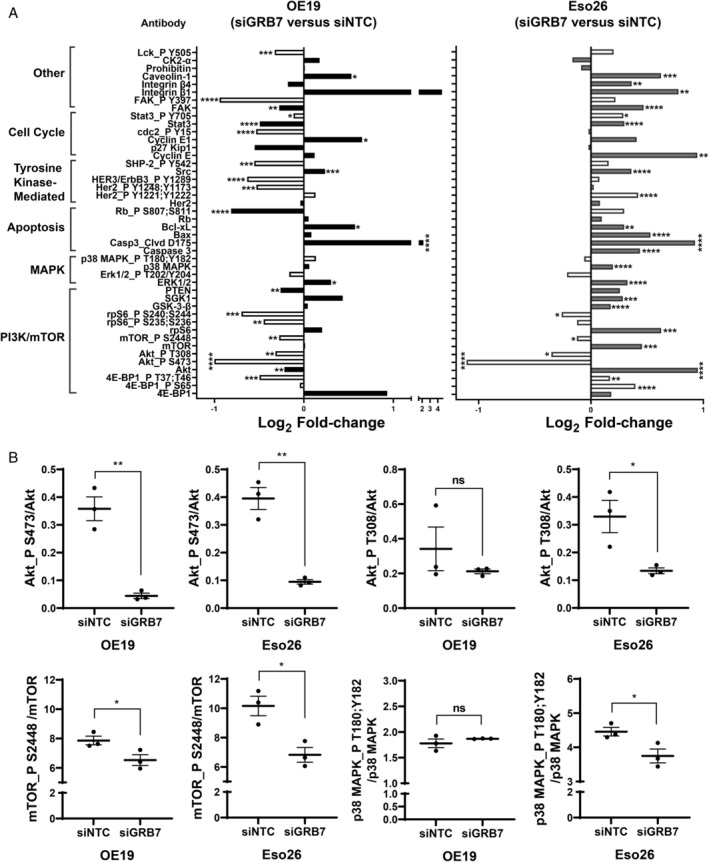
Knockdown of GRB7 in OAC cell lines leads to decreased activation of pro‐growth and survival signalling pathways and upregulation of apoptotic mediators. RPPA analysis was performed on lysates from Eso26 and OE19 OAC cells treated with siGRB7 for 96 h and compared with siRNA control treated cells. (A) Results are presented as log_2_ fold‐change of mean relative fluorescence intensity (RFI) for GRB7 knockdown (siGRB7) compared with control (siNTC) calculated from three biological replicates, each with three to four technical replicates. Positive values represent upregulation and negative values represent downregulation. Unfilled and filled bars represent phosphorylated and total proteins, respectively. (B) Representative graphs of ratio phospho/total protein (RFI) for OE19 and Eso26 cells transfected with siNTC Control or siGRB7 (RPPA). Bars represent mean ± SEM from three independent experiments, Welch's *t*‐test: ns, not significant; *p < 0.05, **p < 0.01, ***p < 0.001, ****p < 0.0001.

To explore the potential mechanism underpinning GRB7 oncogenic signalling, we performed RPPA on protein lysates isolated from OE19 and Eso26 cells transiently treated with siRNA targeting GRB7 or control siRNA (Figure 4 and supplementary material, Figure S3). The RPPA included a total of 45 proteins/phosphoproteins, of which six were upregulated and 19 downregulated in OE19 cells and 22 were upregulated and four downregulated in Eso26 cells following knockdown of GRB7. We looked specifically at the activity of the signalling pathways through detection of phosphorylation of signalling intermediates as well as their total protein. Signalling through the PI3K/mTOR (Akt_P 473, Akt_P308, rsS6_P S240;244 and mTOR_P S2448) and MAPK (Erk1/2_P T202/Y204) pathways was decreased by GRB7 knockdown in both OE19 and Eso26 cells, as displayed by decreased signal intensities in the phospho‐kinases (Figure 4A and supplementary material, Figure S3A,B). In addition, OE19, but not Eso26, cells demonstrated decreased signal intensities in 4E‐BP1_P T37;T46 phosphoprotein. These changes were even more striking when adjusted for total protein (Figure 4B). This suggests reduced signal transduction leading to decreased cellular proliferation, survival, and metabolism, and further supports our hypothesis that GRB7 acts as a central node that mediates oncogenic signalling in OAC.

In contrast, we observed enhanced signalling intensities for mediators of apoptosis, such as cleaved caspase 3 (Casp3_Clvd D175), within Eso26 and OE19 cells upon GRB7 knockdown, demonstrating an essential role of GRB7 signalling in promoting cell survival. Moreover, pro‐apoptotic Bax was increased in Eso26, whereas OE19 cells displayed decreased Rb_P S807;S811, which could contribute to cell cycle arrest and subsequent cell death upon GRB7 knockdown (Figure 4A and supplementary material, Figure S3C). Interestingly, HER2 high‐expressing and trastuzumab‐sensitive OE19 cells displayed decreased phosphorylation of HER2 and HER3 receptors (Her2_P Y1248;Y1173, HER3/ErbB3), SHP‐2 (SHP‐2_P Y542), and cell cycle regulators such as CDC2 (cdc2_P Y15) upon GRB7 knockdown. Contrastingly, Eso26 cells, which are HER2 high‐expressing but insensitive to trastuzumab, exhibited no change of these phosphoproteins and, moreover, increased levels of Her2_P Y1221;Y1222 (Figure 4A and supplementary material, Figure S3D,E).

### GRB7 overexpression results in a cell context‐dependent increase in cell growth and migratory capacities

**Figure 5:**
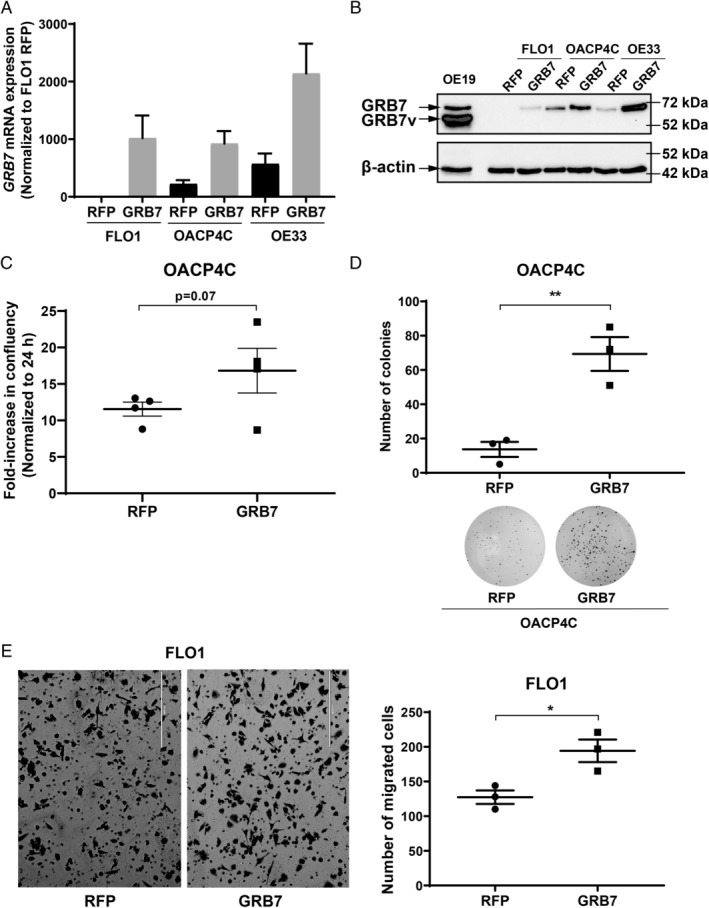
Increased oncogenic potential upon GRB7 overexpression in OAC cell lines. (A)*GRB7* mRNA and (B) protein expression levels following overexpression of GRB7 or control RFP cDNA in FLO1, OACP4C, and OE33 cells. (C) Cell confluency measured at 196 h in OACP4C cells containing GRB7 versus RFP expression vector. Individual data points represent the mean for each experiment. (D) Counts for clonogenic assay of OACP4C cells following GRB7 overexpression versus RFP control (top) and representative image of colony formation (bottom). (E) Representative image (left) and cell count (right) of Boyden chamber migration assay of FLO1 GRB7‐overexpressing cells versus RFP control. Scale bars = 400 μm. Unpaired *t*‐test, *p < 0.05, **p < 0.01; bars represent mean ± SEM for three (D, E) and four (C) independent experiments.

We next examined the consequences of GRB7 overexpression in OAC cell lines with lower endogenous expression of GRB7 (OE33, OACP4C, and FLO1) and in which GRB7 knockdown had no effect on cell growth. We hypothesised that GRB7 overexpression in these cell lines would activate oncogenic signalling and promote tumour phenotypes. Cells were transduced with lentiviral vectors containing GRB7 or control RFP cDNA. GRB7 overexpression (Figure 5A,B) increased cell proliferation in OACP4C cells (Figure 5C) but not in FLO1 and OE33 cells (Figure S4A,B). Consistent with this, GRB7 overexpression significantly increased colony‐forming ability in OACP4C cells (Figure 5D). To extend insights into GRB7 oncogenic function, we performed migration assays in the same cell lines. GRB7 overexpression significantly increased migratory potential in FLO1 (Figure 5E), but not in OACP4C and OE33 cells (data not shown). Together, GRB7 acts as an oncogene and represents a promising therapeutic target in the cells with GRB7 overexpression. In order to pursue GRB7 as a therapeutic target, we further functionally characterised long‐term GRB7 knockdown and its anti‐tumour activity *in vivo*.

### shRNA‐mediated GRB7 knockdown decreased cell proliferation and long‐term cell survival

**Figure 6:**
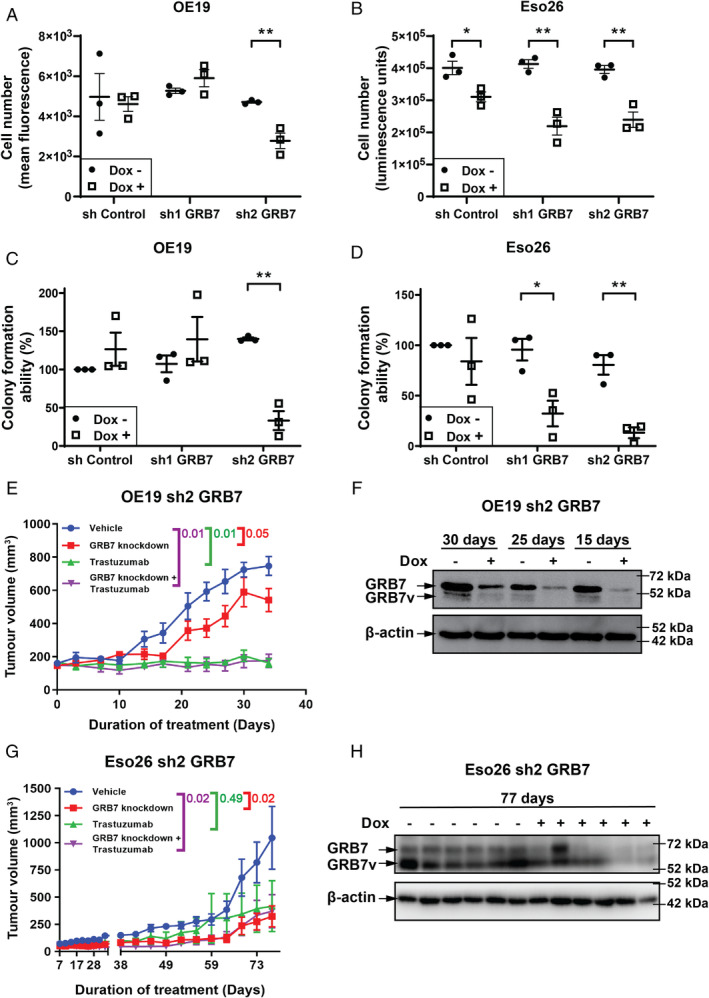
GRB7 knockdown in OAC cell line xenografts attenuates tumour growth. Following initial shRNA induction (72 h 2 μg/ml Dox), cells were replated and the viability of (A) OE19 and (B) Eso26 cells was assessed at 120 h. Quantification of colony formation assays in (C) OE19 and (D) Eso26 cells following induction of sh Control or sh GRB7 relative to sh Control without doxycycline. Bars represent mean ± SEM for three independent experiments. Statistics: (A–D) Unpaired *t*‐test, *p < 0.05, **p < 0.01 compared with no Dox treatment. Growth of (E) OE19 and (G) Eso26 CLXs containing GRB7 sh2 construct in NSG mice treated with vehicle (sterile water), doxycycline (GRB7 knockdown), trastuzumab (10 mg/kg, twice per week) or doxycycline/trastuzumab combination (*n* = 5 or 6 mice per group; data represent mean ± SEM). Red, green, and purple colour coded numbers represent *P* values (CGGC permutation test; http://bioinf.wehi.edu.au/software/compareCurves/) of comparison between vehicle treatment versus GRB7 knockdown, trastuzumab, and combination of GRB7 knockdown + trastuzumab, respectively. Western blot demonstrating the effectiveness of GRB7 knockdown following *in vivo* shRNA doxycycline induction (Dox +) compared with control (Dox −) at (F) 15, 25, and 30 days in OE19 sh2 GRB7 xenografts and (H) 77 days in Eso26 sh2 GRB7 xenografts. Each line represents an independent xenograft.

To investigate the effect of GRB7 knockdown *in vivo*, we cloned four GRB7‐specific shRNAs into a doxycycline‐inducible lentiviral expression vector (supplementary material, Figure S5A). The two most efficient constructs (sh1 and sh2) were chosen for further functional validation and analyses. Expression of sh1 or sh2 RNAs effectively decreased GRB7 protein levels (both full‐length and GRB7 variant protein) in both OE19 and Eso26 cells (supplementary material, Figure S5B,C), but only sh2 decreased cell proliferation in both cell lines (supplementary material, Figure 6A,B). The effectiveness of sh1 GRB7 to inhibit proliferation in Eso26, but not in OE19, cells correlated with higher endogenous and residual GRB7 protein levels following sh1 activation in OE19 cells (supplementary material, Figure S5B). More importantly, consistent with the effects on cell proliferation, GRB7 knockdown using shRNA dramatically reduced colony formation in OE19 cells (Figure 6C) and Eso26 cells (Figure 6D). Taken together, these results demonstrate the importance of GRB7 in cell proliferation and clonogenic capacity in GRB7‐amplified OAC cell lines.

### GRB7 inhibition displays strong anti‐tumour activity in OAC cell line xenografts

We next investigated the anti‐tumour activity of GRB7 inhibition and/or trastuzumab *in vivo* over the time course of the treatment. In OE19 xenografts, tumour growth was completely inhibited by trastuzumab alone or in combination with GRB7 knockdown (Figure 6E). Importantly, GRB7 knockdown alone also impaired tumour growth compared with vehicle treatment. Noticeably, GRB7 knockdown alone initially prevented tumour growth but tumour volume began to increase around 17 days after commencing treatment with doxycycline (Figure 6E). Consistent with this, GRB7 protein expression in tumours showed a gradual increase over time despite continued treatment with doxycycline in a separate cohort of six mice (Figure 6F). This is potentially due to diminished efficacy of the shRNA over time and/or selective outgrowth of clones with higher GRB7 expression. In contrast to the OE19 xenografts, trastuzumab treatment resulted in modest, but not statistically significant tumour growth inhibition of Eso26 tumour xenografts during the full time course of the treatment, while GRB7 knockdown alone or in combination with trastuzumab had significant anti‐tumour activity compared with the vehicle treatment group (Figure 6G,H). As expected, OE19 and Eso26 sh Control xenografts did not respond to doxycycline treatment (supplementary material, Figure S5D,E). Overall, our results showed that GRB7 acts as an oncogene via orchestrating multiple oncogenic signalling pathways. Foremost, GRB7 inhibition has strong anti‐tumour activity and pursuing it in clinical practice as a therapeutic target could provide an advantage in future cancer treatment.

## Discussion

There is an unmet need for new, effective treatments for OAC. Significant focus has been directed towards targeting the *HER2* oncogene in OAC, which is reported to be amplified and overexpressed in approximately 15% of cases [12, 15]. However, this approach has largely failed to replicate the success obtained with anti‐HER2 therapies in HER2‐positive breast cancers, with median overall survival for HER2‐positive gastric or gastro‐oesophageal junction adenocarcinoma patients receiving trastuzumab showing only modest survival benefit [10]. Furthermore, only a proportion of patients with HER2‐positive tumours respond to trastuzumab [14].

We report that *GRB7*, located within the 17q12 amplicon adjacent to *HER2*, is co‐amplified and co‐overexpressed, at both the transcript and the protein levels, with HER2 across a panel of OAC cell lines. More importantly, we report, for the first time, frequent GRB7 overexpression in an OAC patient cohort based on immunohistochemistry. Although a previous study has reported that *HER2* and *GRB7* co‐amplify in OAC samples [32], our data demonstrate that this relationship between HER2 and GRB7 does not translate linearly at the transcript and protein expression levels, suggesting that there are other mechanisms leading to upregulation of GRB7 in OAC. As such, additional mechanisms regulating GRB7 expression via miR‐193a‐3p have been reported in breast [34] and ovarian [35] carcinogenesis. Crucially, patients with GRB7 high‐expressing tumours had a tendency for a shorter overall survival, which could be only partially attributed to co‐amplification of HER2. These data suggest that GRB7 overexpression has a potential oncogenic role in OAC tumourigenesis and is consistent with findings in breast [18] and gastric cancer [36] that support a role for GRB7 in the promotion of cancer growth.

In support of this idea, we show that GRB7 orchestrates OAC tumour cell growth, survival, and migration in cell line models. Although GRB7 has been reported to associate with, and potentially transmit oncogenic signalling through, the HER2 receptor [17], our results indicate that GRB7 plays a role independent of HER2 signalling in OAC. Importantly, aberrantly expressed GRB7 mediates signalling from the numerous receptor tyrosine kinases (RTKs), such as EGFR, HER2, HER3, IR, IGF‐R, PDGFR, and FGFR, that are involved in the control of cellular growth, and FAK and EphB1 regulated migration processes, to downstream signalling cascades [17]. Importantly, our RPPA results provide a mechanistic basis for characterising GRB7 as a mediator and driver of OAC carcinogenesis. As such, GRB7 likely transmits signalling through PI3K/mTOR, MAPK, and RTKs, but also has a role in apoptosis, enabling cancer cells to proliferate and survive. Taken together, the acquisition of the malignant cancer cell phenotype is enhanced with GRB7 overexpression. Thus, from the functionally important GRB7 interaction partners, it is likely that GRB7 is increasing the oncogenic potential of cancer cells by sustaining cell growth, preventing apoptosis, and either acquiring or potentiating migration capabilities. Hence, overexpression of GRB7 in OAC tumours likely provides oncogenic drive in combination with RTK mutational activation or amplification and overexpression, and may even do so without the need for prominent overexpression of upstream oncogenic RTKs. Indeed, detailed research into OAC genetics has revealed that individual OACs exhibit oncogenic activation of multiple RTKs, meaning that using multiple drugs could be required in eradicating cancer [37]. Of importance, as demonstrated in this study, GRB7 functions as an adaptor molecule in multiple oncogenic signalling pathways. Therefore, an alternative may be to use GRB7 as a therapeutic target, as inhibiting GRB7 may weaken cancer cell growth and lead to cell death by blocking multiple oncogenic signalling pathways.

An important finding here is that tumour growth suppression is achieved through depletion of GRB7 in HER2‐ and GRB7‐overexpressed, but trastuzumab‐insensitive, tumour cells. Targeting *GRB7* with RNAi decreased the levels of both full‐length and variant GRB7 protein; therefore we are unable to conclude whether full‐length or the variant protein or both are exerting oncogenic effects. However, ectopic overexpression of full‐length GRB7 alone was sufficient to promote proliferation, colony formation, and migration. Given that many HER2‐overexpressing OACs are intrinsically resistant to HER2 inhibition or acquire resistance following treatment, targeting GRB7 might be a promising therapeutic approach for HER2‐resistant cancers. Furthermore, GRB7 has been characterised as a promising therapeutic target in breast cancer treatment, primarily in combination with either doxorubicin or trastuzumab [38]. Interestingly, our results revealed a significant reduction of OAC cell proliferation following GRB7 depletion, but no synergistic effect between GRB7 depletion and trastuzumab treatment. However, using GRB7 depletion in combination with inhibition of other RTKs might be beneficial and further studies are required.

Given the high toxicity rates in the patients treated with chemotherapy compared with treatment with molecular‐targeted therapies [8], it is rational to utilise highly specific GRB7 inhibitors in patient care. As such, recent efforts to target GRB7 have resulted in the development of bicyclic peptide GRB7‐SH2 inhibitors that have high specificity as well as high binding affinity [39, 40]. In an era of advances in developing molecular‐targeted therapy against SH2 domains in cancers [41], our preclinical studies endorse the future development of GRB7 inhibitors and their potential clinical use in OAC patient treatment.

## Author contributions statement

JRG, DSHL, WAP and NJC designed the overall study. JRG conceived and carried out experiments, analysed data, and wrote the paper. DSHL and JVM conceived and carried out shGRB7 design experiments. MJY conceived and carried out IHC staining experiments. AAM and KJS conceived and carried out RPPA experiments. GDE provided TMAs, clinical data, and survival analysis. JRG, NJC and CM analysed and scored GRB7 staining on TMAs. CPD, JVM, DSHL, WAP, NJC and JRG revised and edited the paper, and all the authors had final approval of the submitted and published versions.

## Supporting information


**Supplementary materials** **and methods**

**Figure S1.** Functional characterisation of GRB7 knockdown in OAC cell lines
**Figure S2.** GRB7 knockdown induces an increase in the subG0/G1 subpopulation of the cell cycle in GRB7 high‐expressing OAC cell lines
**Figure S3.** GRB7 mechanism of action through signalling pathways in OAC
**Figure S4.** Proliferation assay following GRB7 overexpression in OAC cell lines
**Figure S5.** The effect of doxycycline on the growth of OE19 and Eso26 sh Control cell line xenografts

**Table S1.** Antibodies for western blotting and immunohistochemistry
**Table S2.** mRNA target site for siRNA GRB7‐SMART pool
**Table S3.** mRNA target site for shGRB7
**Table S4.** RT‐qPCR primer sequences
**Table S5.** *HER2* (*ERBB2*) and *GRB7* gene copy number (CN) in EAC cell lines
