# Supplementary material for: GRB7 is an oncogenic driver and potential therapeutic target in oesophageal adenocarcinoma
Source: J Pathol. 2020 Sep 15;252(3):317–29. doi: 10.1002/path.5528 (PMC7693356; doi:10.1002/path.5528)
Supplement: Supplementary file 1 — Supplementary materials and methods [file PATH-252-317-s001.docx]

**GRB7 is an oncogenic driver and potential therapeutic target in oesophageal adenocarcinoma**

JR Gotovac *et al. J Pathol* DOI: 10.1002/path.5528

**Supplementary materials and methods**

Reference numbers refer to the main text list

**Histology and immunohistochemistry (IHC)**

The IHC protocol included blocking sections in Dual Endogenous Enzyme Block (Dako, Agilent, Santa Clara, CA, USA) high pH followed by 10% (w/v) bovine serum albumin (BSA) (Sigma-Aldrich, St Louis, Missouri, USA) in Tris-buffered saline containing 0.05% Tween-20 (TBS-T) (Sigma-Aldrich) and incubation with primary anti-GRB7 antibody, 1:500 diluted in 1% (w/v) BSA in TBS-T for 1 h at room temperature, followed by incubation overnight at 4 °C. Primary antibodies were detected using horseradish peroxidase (HRP)-conjugated anti-rabbit secondary antibody polymer (Dako) and visualized with 3,3'-diaminobenzidine (DAB) using the EnVision+ Detection System (DakoCytomation) according to the manufacturer’s protocol, and counterstained with hematoxylin. IHC on positive and negative control tissues was performed concurrently. A list of the antibodies used is provided in supplementary material, Table S1.

**Cell lines and culture**

OE19, OE33, and HEK-293T cells were obtained from the American Type Culture Collection (Manassas, VA, USA). FLO-1, OACM5.1, Eso26, Eso51, SKGT4, and OACP4C cells were kindly gifted by Rebecca Fitzgerald (University of Cambridge, UK). JH-EsoAd1 cells were provided by James Eshleman (Johns Hopkins University, MD, USA) and immortalised normal human oesophageal squamous (NES) epithelial cells were provided by Rhonda Souza (University of Texas, Southwestern Medical Centre, TX, USA). OANC1 cells were developed in our laboratory [42]. The information on the source, tumour location, and original tumour aggressiveness that OAC cell lines have been derived from, has been summarised in previously published work from Boonstra *et al* [43]. Eso51, OE19, OE33, OACM5.1, ESO26, SKGT4, OACP4C, and JH-EsoAd1 cells were cultured in Roswell Park Memorial Institute (RPMI) 1640 medium containing 2.5 mm l-glutamine (Thermo Fisher Scientific, Carlsbad, CA, USA). HEK-293T, FLO-1, and OANC1 cells were grown in Dulbecco’s Modified Eagle’s Medium (DMEM) containing 2.5 mm l-glutamine and 4.5 g/l d-glucose (Thermo Fisher Scientific). NES cells were grown in MCDB-153 medium containing 400 ng/ml hydrocortisone, 20 ng/ml epidermal growth factor (EGF), 20 mg/ml adenine, 140 μg/ml bovine pituitary extract, ITS Liquid Media Supplement (100×) for the final concentration of 2.5 μg/ml insulin, 1.4 μg/ml transferrin, 1.3 ng/ml selenium (Sigma-Aldrich), 10 nm cholera toxin, 5% v/v fetal bovine serum (FBS), 375 ng/ml diflucan, and 4 mm l-glutamine (Glutamax, Thermo Fisher Scientific) and adjusted to a pH of 7.2. All cell lines were grown at 37 °C with 5% CO_2_. Unless otherwise specified, all cell culture media contained 10% v/v FBS, 50 U/ml penicillin, and 50 mg/ml streptomycin (Thermo Fisher Scientific).

**Cellular proliferation assays**

Cellular proliferation was determined using a microscopy-based real-time live cell imaging system that measures cellular confluency (Incucyte FLR, Essen BioScience, Ann Arbor, MI, USA). For these experiments, cells were seeded in 96-well plates with transfection reagents or treated with doxycycline, trastuzumab or vehicle and imaged every 24 h for up to 144 h. For proliferation experiments following GRB7 overexpression, 2 × 10^4^ Flo1, OACP4C, and OE33 cells were seeded in 96-well plates and imaged at the last time point, as indicated.

**Cell viability assays**

Cell viability was assayed using AlamarBlue^®^ (Thermo Fisher Scientific) or CellTiter-Glo^®^ (Promega, Madison, WI, USA) reagents. Following shRNA induction with doxycycline for 72 h, cell viability was assessed at 120 h with the addition of 20% v/v AlamarBlue^®^ (Thermo Fisher Scientific) (OE19 cells) or 50% v/v CellTiter-Glo^®^ (Promega) (Eso26 cells) without removing pre-existing medium in 96-well plates. Fluorescence intensity (AlamarBlue^®^, Thermo Fisher Scientific) was measured using a FLUOstar OPTIMA plate reader (BMG Labtech, Mornington, Australia) at an excitation of 540 nm and an emission of 590 nm following incubation for 2 h at 37 °C. Luminescence (CellTiter-Glo^®^, Promega) was measured using a Cytation 3 Imaging Reader (BioTek, Winooski, VT, USA) upon incubation for 10 min at room temperature.

**Clonogenic survival assay**

To investigate the effects of GRB7 knockdown (shRNA) on long-term survival, cells were induced with 2 µg/ml doxycycline (Merck, Kenilworth, NJ, USA) or vehicle (saline) for 72 h. Then 5 × 10^3^ Eso26 or OE19 cells per well were seeded in six-well plates in media with 2 µg/ml doxycycline or saline. Colonies were allowed to form for 10 days (Eso26) or 14 days (OE19) with addition of medium (5 ml) ± doxycycline after 7 days. To investigate the effect of GRB7 overexpression on colony-forming ability, OACP4C, OE33, and FLO1 cells containing constructs expressing GRB7 or control red fluorescent protein (RFP) were seeded (1 × 10^3^ per well) in six-well plates in 2 ml of medium and allowed to form colonies for 10 days with 2 ml of fresh media added at day 5. Cell colonies were fixed and stained with crystal violet (0.5% w/v). Discrete colonies of more than 50 cells were counted using ImageJ Cell Counter.

**Migration assay**

Flo1, OACP4C, and OE33 cells (5 × 10^5^) expressing GRB7 or RFP in serum-free medium were seeded into the upper chamber of 8 μm pore size Boyden Chambers (Merck) with media containing 10% serum in the lower chamber and incubated at 37 °C with 5% CO_2_ for 24 h. The membranes were fixed with 100% methanol for 15 min at −20 °C and then stained with crystal violet (0.5% w/v). Representative photographs were taken using an AMG EVOS FL microscope (Advanced Microscopy Group, Thermo Fisher Scientific) and cells that had migrated were counted using ImageJ Cell Counter.

**Apoptosis assay**

OE19 and Eso26 cells were seeded at 2.5 × 10^5^ cells per well in six-well plates and cultured following transfection with GRB7 siRNA for 144 h or trastuzumab treatment for 120 h. Cells were stained with Annexin V-FITC antibody and propidium iodide (PI) (BD Pharmingen kit; BD Biosciences, Franklin Lakes, NJ, USA) according to the manufacturer’s instructions. The extent of apoptosis (per cent of Annexin-V-positive cells) was determined by flow cytometry (BD FACSCanto™ II, BD Biosciences) and analysed using Flowlogic software (Inivai Technologies, Mentone, Australia).

**Cell cycle analysis**

OE19 and Eso26 cells at 2.5 × 10^5^ per well were seeded into six-well plates and following transfection with GRB7 siRNA pool, they were cultured for 144 h or subjected to trastuzumab treatment for 120 h. All cells were collected by centrifugation, washed in cold PBS with 1% FBS, fixed in 70% ethanol, and stained for 2 h at room temperature in the dark with 25 µg/ml propidium iodide (Molecular Probes, Eugene, OR, USA) and 40 µg/ml RNAse A (Qiagen, Hilden, Germany) in PBS. A minimum of 10 000 single cell events were detected by flow cytometry (BD FACSCanto™ II, BD Biosciences) and analysed using Flowlogic software (Inivai Technologies).

**Western blotting analysis**

Cells or tissues were lysed at 4 °C in RIPA buffer (1 mm EDTA, 1% v/v NP40, 0.5% w/v sodium deoxycholate, 0.1% w/v SDS, 50 mm sodium fluoride, 1 mm sodium pyrophosphate in PBS; all purchased from either Sigma-Aldrich or Merck) with added phosphatase (PhosphoSTOP, Roche, Basel, Switzerland) and protease (Complete ULTRA, Roche) inhibitors according to the manufacturer’s guidelines. Cells were harvested at the indicated time points following transfection with siRNA, or treatment with 2 µg/ml doxycycline or vehicle (for shRNA experiments). Tumour tissues were harvested from mice at the indicated time points and homogenised with the PowerLyser^TM^ 24 (MO BIO Laboratories, Carlsbad, CA, USA) in tubes containing metallic beads.

Protein concentrations were quantified using a Bradford protein assay (BioRad Laboratories, Hercules, CA, USA). Equivalent amounts of protein lysates were boiled in sample buffer [313 mm Tris–HCl, pH 6.8; 50% (v/v) glycerol; 10% (v/v) β-mercaptoethanol; 10% (w/v) SDS, 0.05% (w/v) bromophenol blue], resolved by SDS-PAGE using the range of 7–15% w/v acrylamide gels according to the size of the protein of interest, and transferred to polyvinylidene difluoride membranes. Membranes were blocked for 1 h in buffer containing 5% w/v skim milk powder, 0.1% v/v Tween 20 in TBS, and probed overnight at 4 °C with the primary antibody (supplementary material, Table S1). Blots were washed three times in rinsing buffer (0.1% v/v Tween 20 in TBS) for 10 min, followed by incubation with horseradish peroxidase-conjugated secondary antibody (Dako, Agilent) for 1 h at room temperature. Proteins were visualised using Western Lightening Enhanced Chemiluminescence (ECL) (PerkinElmer, Waltham, MA, USA) or ECL Plus Western blotting substrate kit (Thermo Fisher Scientific). Blots were reprobed with anti-β-actin or anti-GAPDH antibody to determine protein loading.

**Reverse phase protein array (RPPA)**

Due to low yield, samples including OE19 cells treated with siGRB7 were concentrated using the Amicon Ultra-0.5 Centrifugal Filter Unit (UFC5003, Merck). Using a Sciclone/Caliper ALH3000 liquid handling robot (Perkin Elmer), samples were prepared in four dilutions (100%, 63%, 40%, and 25%) in 10% CLB1:90% CSBL1 buffer (Zeptosens/Bayer, Leverkusen, Germany) and spotted onto ZeptoChips (Zeptosens/Bayer) in three or four technical replicates using a Nano-plotter-NP2.1 non-contact microarray system (GeSim, Radeberg, Germany). Chips were blocked for 1 h with BB1 buffer (Zeptosens/Bayer), incubated with pre-validated primary antibodies (1:500, 20 h), and Alexa Fluor^®^ 647 anti-rabbit secondary antibody (1:1000, 4 h) (#Z25308, Thermo Fisher Scientific). Chips were read on a Zeptosens instrument and software version 3.1 was used to calculate the relative fluorescence intensity (RFI). All samples were normalised to the background values reported in the secondary antibody-only negative control.
