# Supplementary material for: GRB7 is an oncogenic driver and potential therapeutic target in oesophageal adenocarcinoma
Source: J Pathol. 2020 Sep 15;252(3):317–29. doi: 10.1002/path.5528 (PMC7693356; doi:10.1002/path.5528)
Supplement: Supplementary file 2 — Figure S1. Functional characterisation of GRB7 knockdown in OAC cell lines Figure S2. GRB7 knockdown induces an increase in the subG0/G1 subpopulation of the cell cycle in GRB7 high‐expressing OAC cell lines Figure S3. GRB7 mechanism of action through signalling pathways in OAC Figure S4. Proliferation assay following GRB7 overexpression in OAC cell lines Figure S5. The effect of doxycycline on the growth of OE19 and Eso26 sh Control cell line xenografts [file PATH-252-317-s002.docx]

**GRB7 is an oncogenic driver and potential therapeutic target in oesophageal adenocarcinoma**

JR Gotovac *et al. J Pathol* DOI: 10.1002/path.5528

**Supplementary Figures S1–S5**


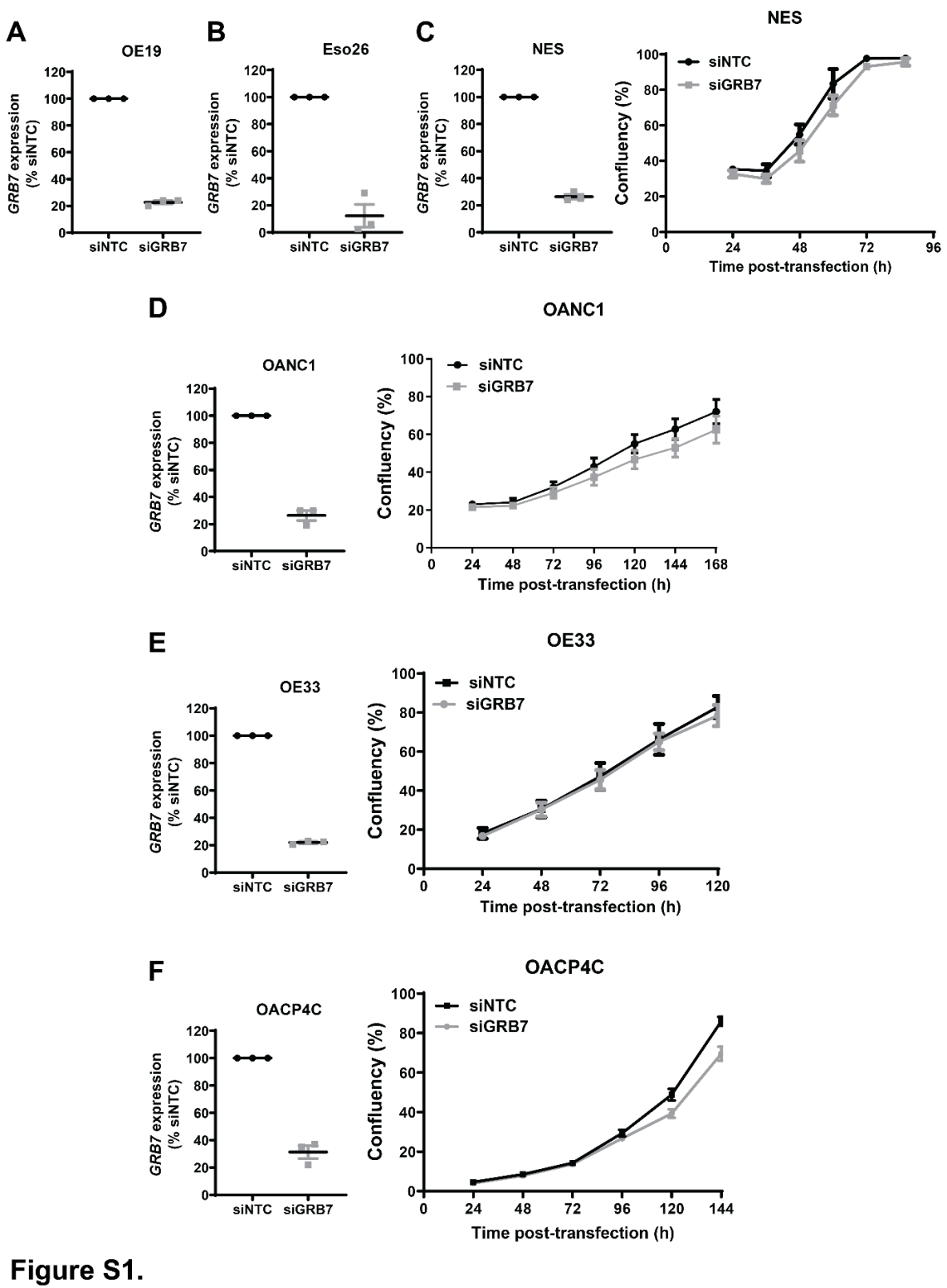


**Figure S1.** **Functional characterisation of GRB7 knockdown in OAC cell lines.** (A, B) *GRB7* mRNA in OE19 and Eso26 cells following knockdown with GRB7 siRNAs (siGRB7) compared with non-targeting control siRNA (siNTC). Expression of mRNA was determined by RT-qPCR 24 h after transfection with siRNA. (C–F) *GRB7* transcript levels following siRNA knockdown (left panels, RT-qPCR) and subsequent time course proliferation assay (right panels, Incucyte) in NES, OANC1, OE33, and OACP4C, respectively. For all experiments, data represent mean ± SEM from three independent experiments (except RT-qPCR assay for OE33 cells, which shows mean ± SEM for technical replicates from one experiment).


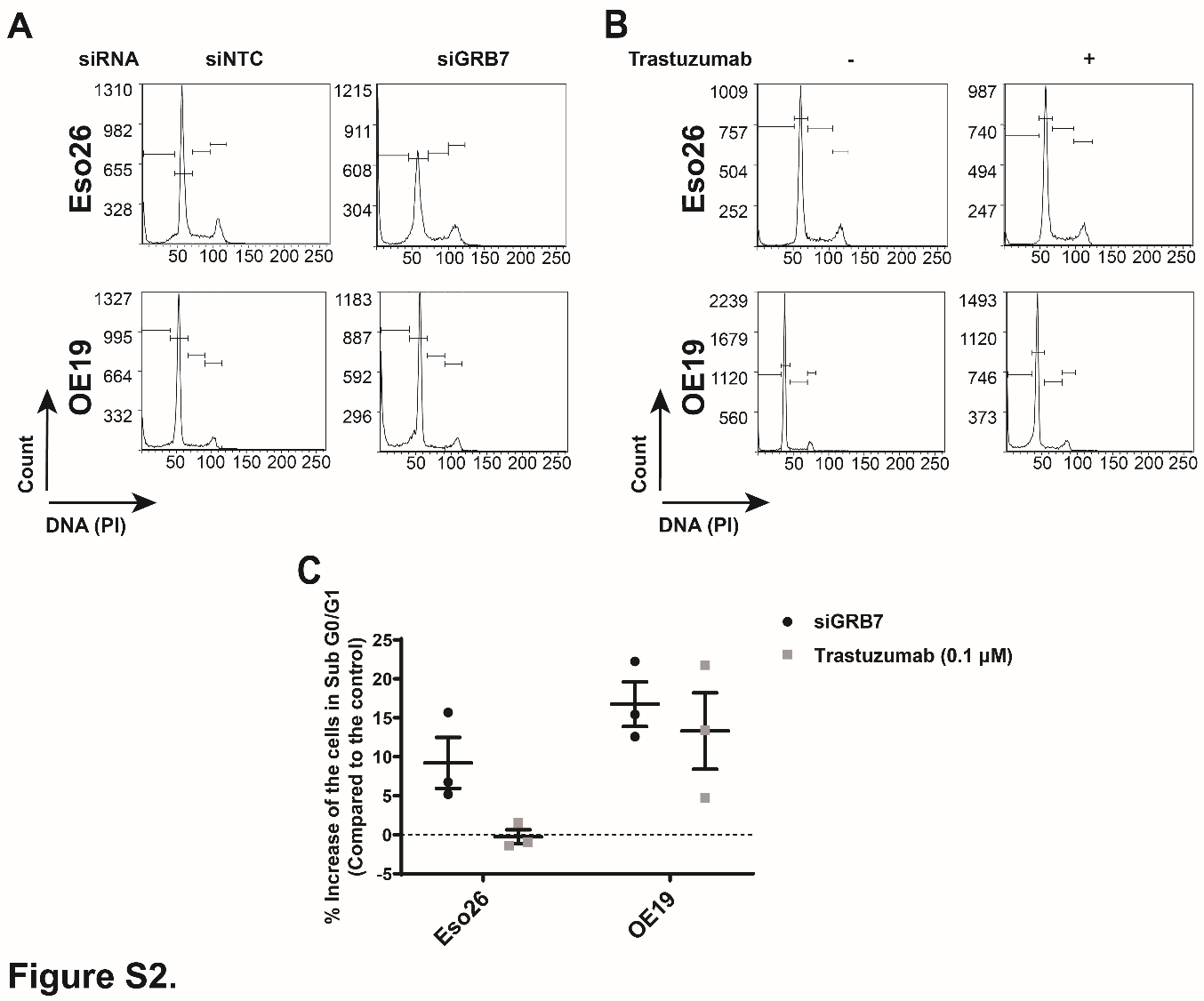


**Figure S2.** **GRB7 knockdown induces an increase in the subG0/G1 subpopulation of the cell cycle in GRB7 high-expressing OAC cell lines.** Representative DNA (propidium iodide, PI) histograms of cell cycle analyses in Eso26 and OE19 cell lines treated with (A) siGRB7 for 144 h or (B) 0.1 µm Trastuzumab for 120 h compared with siNTC or vehicle, respectively. (C) Percentage increase of the subG0/G1 phase of the cell cycle. Bars represent mean ± SEM from three independent experiments.


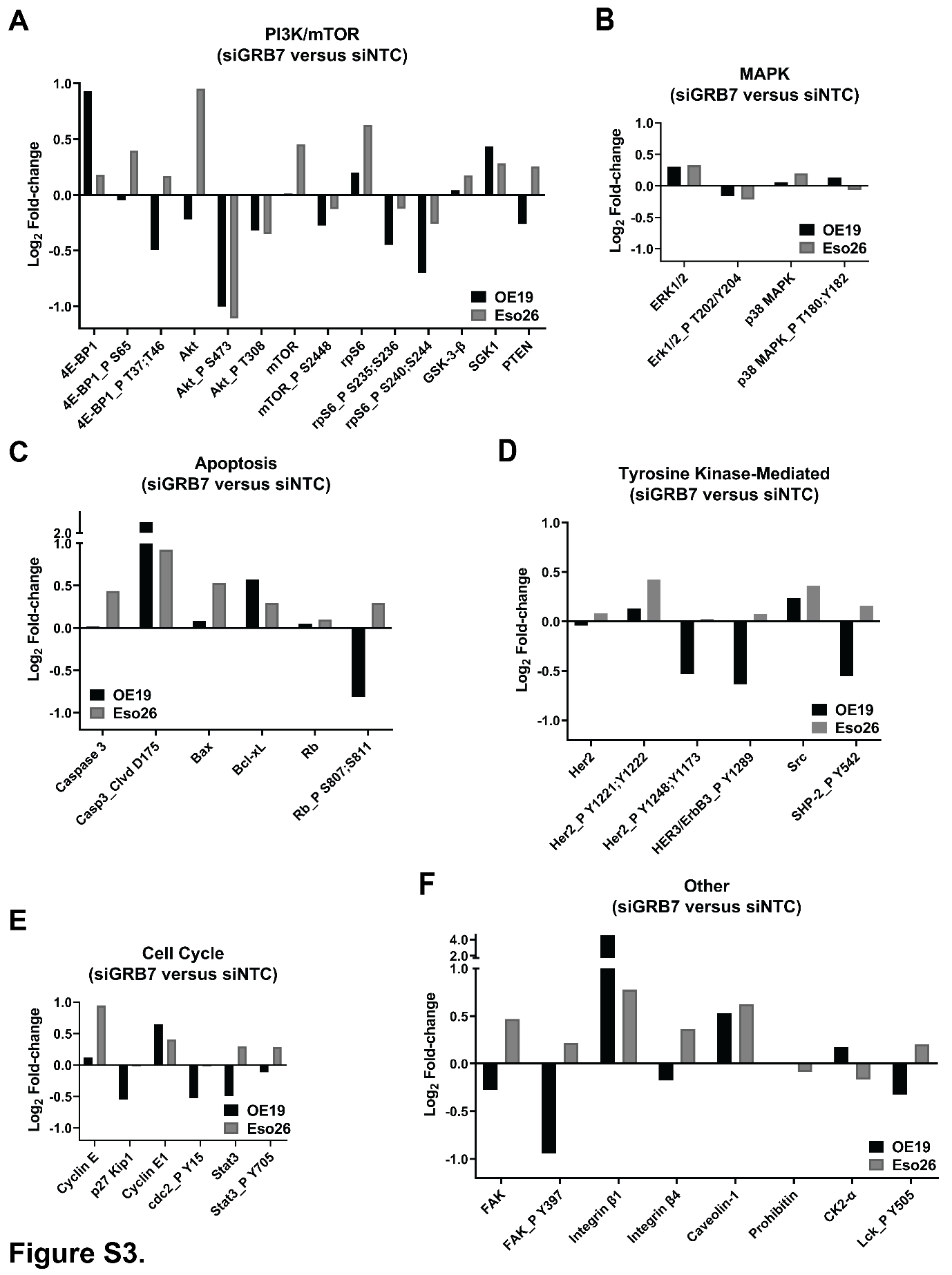


**Figure S3. GRB7 mechanism of action through signalling pathways in OAC.** RPPA analyses of mediators involved in (A) PI3K/mTOR, (B) MAPK, (C) apoptosis, (D) tyrosine kinase, (E) cell cycle, and (F) other signalling pathways in OE19 and Eso26 OAC cells upon GRB7 knockdown. Data represent log_2_ fold-change of RFI values.


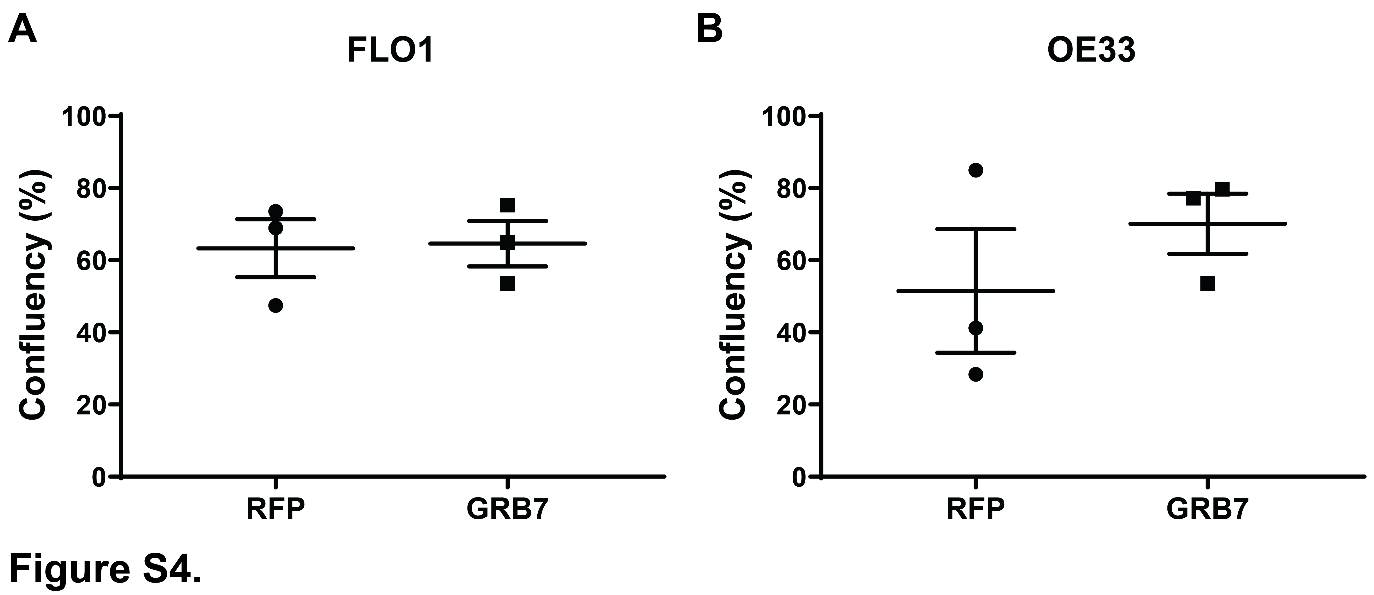


**Figure S4.** **Proliferation assay following GRB7 overexpression in OAC cell lines.** (A) Confluency was measured at 144 h following plating of FLO1 cells and (B) at 96 h following plating of OE33 cells. Bars represent mean ± SEM from three independent experiments.


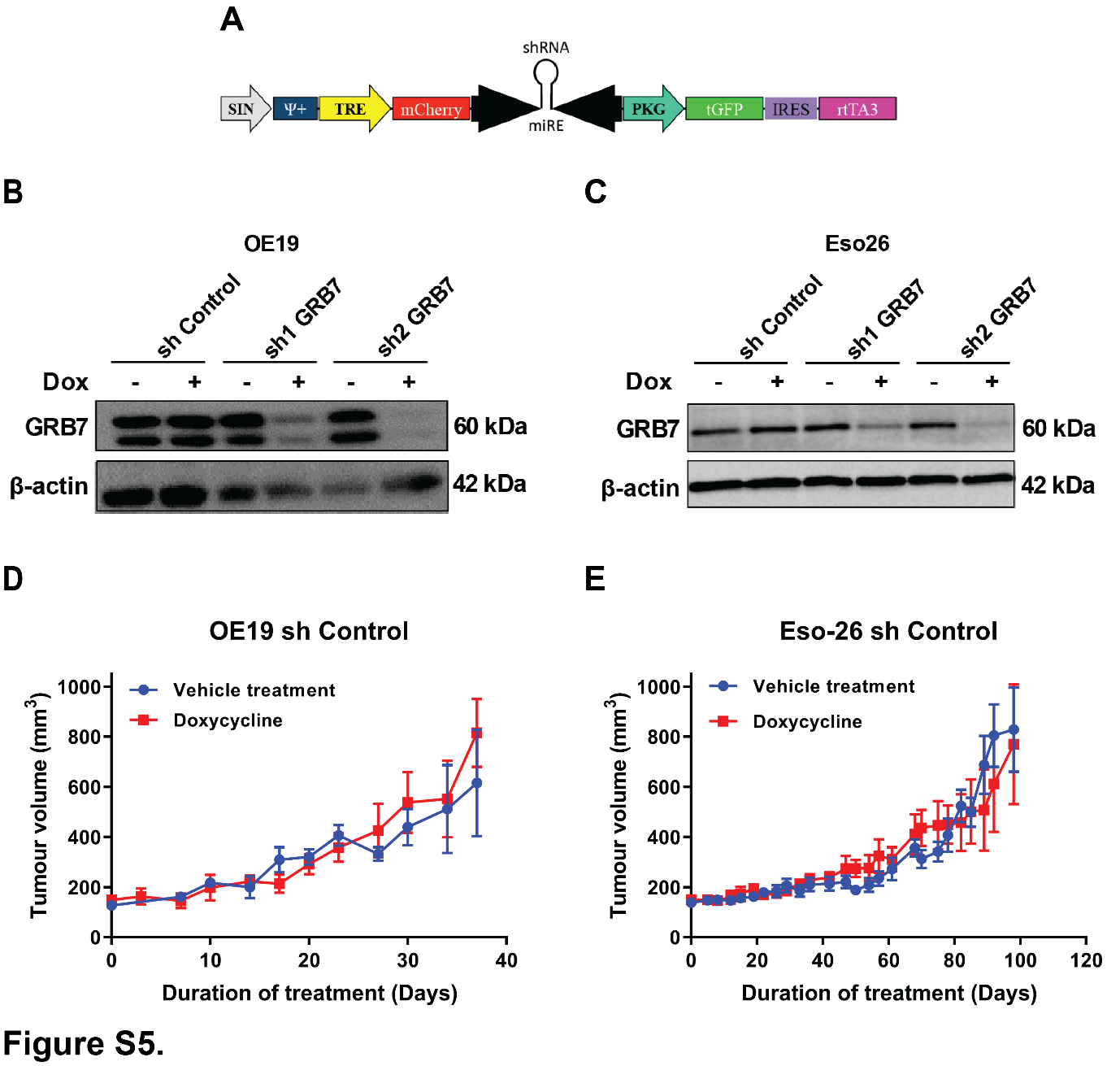


**Figure S5. The effect of doxycycline on the growth of OE19 and Eso26 sh Control cell line xenografts.** (A) Diagram of the LT3GECIR lentiviral doxycycline (Dox)-inducible expression vector with possibility to clone either GRB7-specific shRNA (sh1 or sh2 GRB7) or scramble shRNA control (sh Control). (B, C) The effect of inducible GRB7 shRNA expression for 72 h [2 µg/ml doxycycline (Dox)] on GRB7 protein levels was assessed by western blotting in OE19 and Eso26 cells. (D, E) The effect of doxycycline in chow (600 mg/kg) and water (2 mg/ml) on the growth of (D) OE19 and (E) Eso26 sh Control cell line xenografts. Growth curves represent the average tumour volume for three and six mice used per group in OE19 and Eso26 xenografts, respectively.
