# Supplementary material for: GRB7 is an oncogenic driver and potential therapeutic target in oesophageal adenocarcinoma
Source: J Pathol. 2020 Sep 15;252(3):317–29. doi: 10.1002/path.5528 (PMC7693356; doi:10.1002/path.5528)
Supplement: Supplementary file 3 — Table S1. Antibodies for western blotting and immunohistochemistry Table S2. mRNA target site for siRNA GRB7‐SMART pool Table S3. mRNA target site for shGRB7 Table S4. RT‐qPCR primer sequences Table S5. HER2 (ERBB2) and GRB7 gene copy number (CN) in EAC cell lines [file PATH-252-317-s003.docx]

**GRB7 is an oncogenic driver and potential therapeutic target in oesophageal adenocarcinoma**

JR Gotovac *et al. J Pathol* DOI: 10.1002/path.5528

**Supplementary Tables S1–S5**

**Table S1.** Antibodies for western blotting and immunohistochemistry

| Antibody | Origin | Clone | Source | Use* and (dilution) |
| --- | --- | --- | --- | --- |
| Anti-GRB7 | Rabbit | EPR4378 | LS-C138038, LSbio, Seattle, WA, USA | WB (1:1000) and IHC (1:500) |
| Anti-HER2 | Mouse | 3B5 | Calbiochem, Merck, Kenilworth, NJ, USA | WB (1:1000) |
| Anti-β-actin | Mouse | C4 | MP-Biomedical, Irvine, CA, USA | WB (1:10 000) |
| Anti-GAPDH | Mouse | 6C5 | Abcam, Cambridge, UK | WB (1:10 000) |

*WB = western blotting; IHC = immunohistochemistry.

**Table S2.** mRNA target site for siRNA GRB7-SMART pool

| **siRNA code** | **Target sequence** |
| --- | --- |
| D-012701-01 | AGAAGUGCCUCAGAUAAUA |
| D-012701-02 | UAGUAAAGGUGUACAGUGA |
| D-012701-03 | UGCAGAAAGUGAAGCAUUA |
| D-012701-04 | GGAGAUAGCCGCUUCGUCU |

**Table S3.** mRNA target site for shGRB7

| **Name** | **Sequence** |
| --- | --- |
| hGrb7.211 (sh1) | GGAUCUGUCUCCACCUCAUCU |
| hGrb7.504 (sh2) | CCCAUGUAGUAAAGGUGUACA |
| hGrb7.1407 (sh3) | GGAGGAAGAAGACAAACCACC |
| hGrb7.1101 (sh4) | GUUUCUGUGUCAAGCCCAACA |

**Table S4.** RT-qPCR primer sequences

| **Gene symbol** | **Forward (5'-3')** | **Reverse (5'-3')** |
| --- | --- | --- |
| GRB7 | GCCTTCCGCCTCTTCAAGTA | CACTTCTCAAGGGTGGGGAG |
| HER2 | TTGAGTCCATGCCCAATCC | GCTGTGTTCCATCCTCTGCT |

**Table S5.** *HER2* (*ERBB2*) and *GRB7* gene copy number (CN) in EAC cell lines

| Cell line | CN ERBB2 | Signal | CN GRB7 | Signal |
| --- | --- | --- | --- | --- |
| OE19 | 14 | Gain | 14 | Gain |
| OE33 | 14 | Gain | 14 | Gain |
| OACP4C | 9 | Gain | 9 | Gain |
| Eso26 | 8 | Gain | 8 | Gain |
| OACM5.1 | 2 | No | 2 | No |
| Eso51 | 2 | No | 2 | No |
| SKGT4 | 2 | No | 2 | No |
| FLO1 | 2 | No | 2 | No |

CN = gene copy number. Data obtained from canSAR database (http://cansarblack.icr.ac.uk).
